# *N*′-(2-Fluoro­benzyl­idene)acetohydrazide

**DOI:** 10.1107/S1600536810010627

**Published:** 2010-03-27

**Authors:** Jie Yang, Zhao-De Jiang, Fu-Gong Zhang, Fang-Fang Jian

**Affiliations:** a Microscale Science Institute, Weifang University, Weifang 261061, People’s Republic of China; b Yantai Vocational and Technical institute of Engineering, Yantai 264006, People’s Republic of China; c Ministry of Personnel, Weifang University, Weifang 261061, People’s Republic of China

## Abstract

The title compound, C_9_H_9_FN_2_O, was prepared by the reaction between 2-fluoro­benzophenone and acetohydrazide. In the crystal structure, inversion dimers linked by pairs of N—H⋯O hydrogen bonds occur, generating *R*_2_^2^(8) loops.

## Related literature

For background to Schiff bases, see: Cimerman *et al.* (1997 ▶); For related structures, see: Girgis (2006 ▶); Li & Jian (2008 ▶).
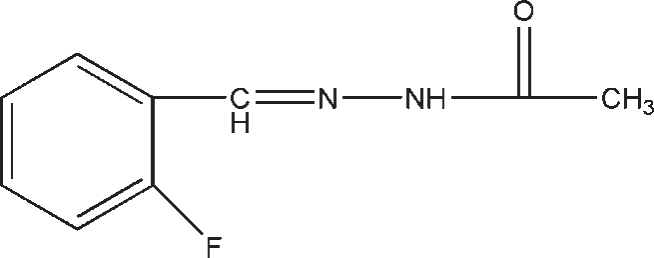


## Experimental

### 

#### Crystal data

C_9_H_9_FN_2_O
*M* *_r_* = 180.18
Monoclinic, 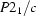

*a* = 5.3227 (11) Å
*b* = 8.4603 (17) Å
*c* = 19.656 (4) Å
β = 93.70 (3)°
*V* = 883.3 (3) Å^3^
*Z* = 4
Mo *K*α radiation
μ = 0.11 mm^−1^
*T* = 293 K
0.30 × 0.30 × 0.20 mm

#### Data collection

Bruker SMART CCD diffractometer
7687 measured reflections
2010 independent reflections
1515 reflections with *I* > 2σ(*I*)
*R*_int_ = 0.036

#### Refinement

*R*[*F* ^2^ > 2σ(*F* ^2^)] = 0.042
*wR*(*F* ^2^) = 0.128
*S* = 1.04
2010 reflections
119 parameters
H-atom parameters constrained
Δρ_max_ = 0.14 e Å^−3^
Δρ_min_ = −0.21 e Å^−3^

### 

Data collection: *SMART* (Bruker, 1997 ▶); cell refinement: *SAINT* (Bruker, 1997 ▶); data reduction: *SAINT*; program(s) used to solve structure: *SHELXS97* (Sheldrick, 2008 ▶); program(s) used to refine structure: *SHELXL97* (Sheldrick, 2008 ▶); molecular graphics: *SHELXTL* (Sheldrick, 2008 ▶); software used to prepare material for publication: *SHELXTL*.

## Supplementary Material

Crystal structure: contains datablocks global, I. DOI: 10.1107/S1600536810010627/hb5371sup1.cif

Structure factors: contains datablocks I. DOI: 10.1107/S1600536810010627/hb5371Isup2.hkl

Additional supplementary materials:  crystallographic information; 3D view; checkCIF report

## Figures and Tables

**Table 1:** Hydrogen-bond geometry (Å, °)

*D*—H⋯*A*	*D*—H	H⋯*A*	*D*⋯*A*	*D*—H⋯*A*
N1—H1*A*⋯O1^i^	0.86	2.08	2.915 (2)	163

Symmetry code: (i) 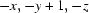.

**Fig. 1.**
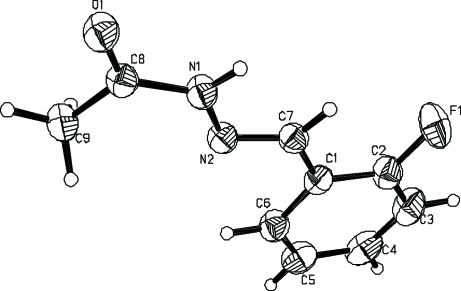
The molecular structure of (I) with displacement ellipsoids drawn at the 30% probability level.

## supplementary crystallographic information

### Comment

Schiff bases have important applications in analytical chemistry (Cimerman *et al.*, 1997). As part of our search for new Schiff bases with similar applications, we synthesized the title compound, (I), and report its crystal structure herein (Fig. 1).

All the bond lengths and angles in (I) are within normal ranges (Li & Jian, 2008). The C7=N2 bond length of 1.2732 (18)Å is slight shorter than the C=N double bond [1.281 (2) Å] reported (Girgis, 2006) in a related compound.

In the crystal structure, adjacent molecules are linked into a centro-symmetric supra-molecular dimer by intermolecular N—H···O hydrogen bonding (Table 1).

### Experimental

A mixture of 2-fluorobenzophenone (0.05 mol) and acethydrazide (0.05 mol) was stirred in refluxing ethanol(30 ml) for 4 h to afford the title compound (yield 70%).  Colourless blocks of (I) were obtained by recrystallization from ethanol at room temperature.

### Refinement

All H atoms were fixed geometrically and allowed to ride on their attached atoms, with C—H and N—H distances in the range 0.93-0.97Å and 0.86 Å, respectively, and with *U*_iso_(H) = 1.2*U*_eq_ of the parent atoms.

### Crystal data

**Table:**

C_9_H_9_FN_2_O	*F*(000) = 376
*M**_r_* = 180.18	*D*_x_ = 1.355 Mg m^−^^3^
Monoclinic, *P*2_1_/*c*	Mo *K*α radiation, λ = 0.71073 Å
Hall symbol: -P 2ybc	Cell parameters from 1515 reflections
*a* = 5.3227 (11) Å	θ = 3.2–27.5°
*b* = 8.4603 (17) Å	µ = 0.11 mm^−^^1^
*c* = 19.656 (4) Å	*T* = 293 K
β = 93.70 (3)°	Block, colourless
*V* = 883.3 (3) Å^3^	0.30 × 0.30 × 0.20 mm
*Z* = 4	

### Data collection

**Table:**

Bruker SMART CCD diffractometer	1515 reflections with *I* > 2σ(*I*)
Radiation source: fine-focus sealed tube	*R*_int_ = 0.036
graphite	θ_max_ = 27.5°, θ_min_ = 3.2°
ω scans	*h* = −6→6
7687 measured reflections	*k* = −9→10
2010 independent reflections	*l* = −25→25

### Refinement

**Table:**

Refinement on *F*^2^	Secondary atom site location: difference Fourier map
Least-squares matrix: full	Hydrogen site location: inferred from neighbouring sites
*R*[*F*^2^ > 2σ(*F*^2^)] = 0.042	H-atom parameters constrained
*wR*(*F*^2^) = 0.128	*w* = 1/[σ^2^(*F*_o_^2^) + (0.0696*P*)^2^ + 0.0888*P*] where *P* = (*F*_o_^2^ + 2*F*_c_^2^)/3
*S* = 1.04	(Δ/σ)_max_ < 0.001
2010 reflections	Δρ_max_ = 0.14 e Å^−^^3^
119 parameters	Δρ_min_ = −0.21 e Å^−^^3^
0 restraints	Extinction correction: *SHELXL97* (Sheldrick, 2008), Fc^*^=kFc[1+0.001xFc^2^λ^3^/sin(2θ)]^-1/4^
Primary atom site location: structure-invariant direct methods	Extinction coefficient: 0.035 (6)

### Special details

**Table:**

Geometry. All esds (except the esd in the dihedral angle between two l.s. planes) are estimated using the full covariance matrix. The cell esds are taken into account individually in the estimation of esds in distances, angles and torsion angles; correlations between esds in cell parameters are only used when they are defined by crystal symmetry. An approximate (isotropic) treatment of cell esds is used for estimating esds involving l.s. planes.
Refinement. Refinement of *F*^2^ against ALL reflections. The weighted *R*-factor wR and goodness of fit *S* are based on *F*^2^, conventional *R*-factors *R* are based on *F*, with *F* set to zero for negative *F*^2^. The threshold expression of *F*^2^ > σ(*F*^2^) is used only for calculating *R*-factors(gt) etc. and is not relevant to the choice of reflections for refinement. *R*-factors based on *F*^2^ are statistically about twice as large as those based on *F*, and *R*- factors based on ALL data will be even larger.

### Fractional atomic coordinates and isotropic or equivalent isotropic displacement parameters (Å^2^)

**Table:**

	*x*	*y*	*z*	*U*_iso_*/*U*_eq_	
N2	0.4557 (2)	0.26752 (14)	0.01843 (6)	0.0474 (3)	
O1	0.04461 (18)	0.48232 (13)	−0.09198 (5)	0.0578 (3)	
N1	0.2546 (2)	0.35774 (14)	−0.00633 (6)	0.0505 (3)	
H1A	0.1453	0.3885	0.0212	0.061*	
C1	0.6693 (3)	0.15209 (16)	0.11622 (7)	0.0479 (3)	
C8	0.2245 (2)	0.39930 (17)	−0.07282 (7)	0.0473 (3)	
C7	0.4679 (3)	0.24649 (16)	0.08270 (7)	0.0477 (3)	
H7A	0.3469	0.2919	0.1086	0.057*	
F1	0.5354 (2)	0.23104 (15)	0.22231 (5)	0.0865 (4)	
C9	0.4142 (3)	0.3418 (2)	−0.11987 (7)	0.0583 (4)	
H9A	0.3699	0.3789	−0.1652	0.087*	
H9B	0.4168	0.2284	−0.1197	0.087*	
H9C	0.5777	0.3812	−0.1049	0.087*	
C6	0.8398 (3)	0.0646 (2)	0.08064 (8)	0.0592 (4)	
H6A	0.8267	0.0649	0.0332	0.071*	
C2	0.6989 (3)	0.1453 (2)	0.18648 (7)	0.0586 (4)	
C5	1.0271 (3)	−0.0222 (2)	0.11457 (10)	0.0708 (5)	
H5A	1.1397	−0.0794	0.0900	0.085*	
C3	0.8824 (4)	0.0594 (2)	0.22135 (9)	0.0756 (5)	
H3A	0.8947	0.0580	0.2688	0.091*	
C4	1.0489 (3)	−0.0250 (2)	0.18462 (10)	0.0756 (5)	
H4A	1.1760	−0.0839	0.2072	0.091*	

### Atomic displacement parameters (Å^2^)

**Table:**

	*U*^11^	*U*^22^	*U*^33^	*U*^12^	*U*^13^	*U*^23^
N2	0.0448 (6)	0.0508 (6)	0.0460 (6)	0.0032 (5)	−0.0023 (5)	0.0011 (5)
O1	0.0518 (6)	0.0700 (7)	0.0507 (6)	0.0133 (5)	−0.0049 (4)	0.0030 (5)
N1	0.0475 (6)	0.0591 (7)	0.0447 (6)	0.0106 (5)	0.0006 (5)	0.0019 (5)
C1	0.0475 (7)	0.0482 (7)	0.0471 (7)	−0.0069 (6)	−0.0046 (5)	0.0041 (5)
C8	0.0434 (7)	0.0520 (7)	0.0455 (7)	−0.0015 (6)	−0.0035 (5)	−0.0004 (5)
C7	0.0482 (7)	0.0490 (7)	0.0455 (7)	−0.0010 (6)	0.0006 (5)	−0.0021 (5)
F1	0.0899 (8)	0.1230 (10)	0.0462 (5)	0.0075 (6)	0.0006 (5)	−0.0129 (5)
C9	0.0538 (8)	0.0733 (10)	0.0478 (7)	0.0058 (7)	0.0039 (6)	0.0000 (7)
C6	0.0601 (9)	0.0623 (9)	0.0545 (8)	0.0069 (7)	−0.0007 (6)	0.0061 (7)
C2	0.0588 (9)	0.0683 (10)	0.0475 (7)	−0.0101 (7)	−0.0058 (6)	0.0002 (6)
C5	0.0599 (10)	0.0664 (11)	0.0850 (12)	0.0100 (8)	−0.0034 (8)	0.0118 (9)
C3	0.0764 (11)	0.0912 (13)	0.0558 (9)	−0.0153 (10)	−0.0222 (8)	0.0153 (8)
C4	0.0619 (10)	0.0734 (12)	0.0880 (12)	−0.0051 (8)	−0.0233 (9)	0.0253 (10)

### Geometric parameters (Å, °)

**Table:**

N2—C7	1.2732 (18)	C9—H9A	0.9600
N2—N1	1.3775 (16)	C9—H9B	0.9600
O1—C8	1.2269 (17)	C9—H9C	0.9600
N1—C8	1.3529 (17)	C6—C5	1.376 (2)
N1—H1A	0.8600	C6—H6A	0.9300
C1—C2	1.3809 (19)	C2—C3	1.366 (2)
C1—C6	1.394 (2)	C5—C4	1.375 (3)
C1—C7	1.4595 (19)	C5—H5A	0.9300
C8—C9	1.494 (2)	C3—C4	1.379 (3)
C7—H7A	0.9300	C3—H3A	0.9300
F1—C2	1.363 (2)	C4—H4A	0.9300
			
C7—N2—N1	114.63 (11)	H9A—C9—H9C	109.5
C8—N1—N2	121.75 (11)	H9B—C9—H9C	109.5
C8—N1—H1A	119.1	C5—C6—C1	121.01 (15)
N2—N1—H1A	119.1	C5—C6—H6A	119.5
C2—C1—C6	116.37 (14)	C1—C6—H6A	119.5
C2—C1—C7	120.46 (13)	F1—C2—C3	118.90 (14)
C6—C1—C7	123.17 (12)	F1—C2—C1	117.35 (14)
O1—C8—N1	119.12 (13)	C3—C2—C1	123.74 (16)
O1—C8—C9	122.79 (13)	C4—C5—C6	120.45 (17)
N1—C8—C9	118.08 (12)	C4—C5—H5A	119.8
N2—C7—C1	120.89 (13)	C6—C5—H5A	119.8
N2—C7—H7A	119.6	C2—C3—C4	118.43 (16)
C1—C7—H7A	119.6	C2—C3—H3A	120.8
C8—C9—H9A	109.5	C4—C3—H3A	120.8
C8—C9—H9B	109.5	C5—C4—C3	120.00 (16)
H9A—C9—H9B	109.5	C5—C4—H4A	120.0
C8—C9—H9C	109.5	C3—C4—H4A	120.0

### Hydrogen-bond geometry (Å, °)

**Table:**

*D*—H···*A*	*D*—H	H···*A*	*D*···*A*	*D*—H···*A*
N1—H1A···O1^i^	0.86	2.08	2.915 (2)	163

Symmetry codes: (i) −*x*, −*y*+1, −*z*.
